# Supplementary material for: Smoking Cessation Advisors’ Perspectives on Pregnant Women’s Attitudes on the Risks and Consequences of Smoking While Pregnant
Source: Healthcare (Basel). 2025 Nov 22;13(23):3018. doi: 10.3390/healthcare13233018 (PMC12692561; doi:10.3390/healthcare13233018)
Supplement: Supplementary file 1 [file healthcare-13-03018-s001.zip › File S1 Focus Group Topic Guide ¿C Smoking Cessation Advisors.pdf]

File S1: Focus Group Topic Guide – Smoking Cessation Advisors

**Question / Topic**	**Prompts / Probes**	**Notes for Moderator**
**1. Clients' opinions on smoking during pregnancy**	- How aware are clients of the risks to their baby? - Do they mention personal/family experiences when discussing risks? - Differences by age, parity, socioeconomic status? - How do clients express ambivalence or denial? - Examples of justifications for continuing to smoke	Encourage concrete examples and anecdotes. Explore patterns across clients. Avoid leading responses.
**2. Advisors' perception of discussing risks**	- How do you approach the topic with clients? - Difficult or sensitive topics? - Clients' reactions when risks are raised? - Does discussing risks motivate change or create resistance? - Health vs. social/family consequences	Observe differences between advisors' perceptions and actual client responses. Probe for challenges and strategies.
**3. Managing client responses**	- How do you respond to denial or minimization of risks? - Strategies for maintaining rapport while encouraging change? - Handling resistant or defensive clients? - Examples of challenges and resolutions? - Involvement of partners, family, or social support?	Prompt for detailed examples of tactics used. Explore problem-solving strategies.
**4. Methods and tactics used**	- Behaviour change techniques used (goal setting, feedback, motivational interviewing)? - Tailoring approaches to individual beliefs, emotions, or social context? - Useful tools or resources? - Follow-up strategies over multiple sessions? - Techniques most effective for long-term cessation	Explore which strategies are most and least effective. Probe for variation across clients.
**5. Emotional responses and coping**	- Typical emotional responses when discussing risks? - Effects on motivation to quit? - Advisor responses to client emotions	Highlight how advisors handle sensitive emotional issues.
**6. Social influences and support**	- Role of partners, family, or friends in clients' smoking behavior? - Strategies to involve support network? - Impact of social environment on cessation	Explore influence of social context on client success.
**7. Barriers to behavior change**	- Main barriers preventing clients from quitting? - Financial, habitual, or environmental factors? - Normalization of risks based on personal experience?	Encourage discussion of both common and unusual barriers.
**8. Success strategies and motivators**	- Which strategies work best? - Tailoring approaches for individual clients? - Tracking or follow-up over multiple sessions?	Explore patterns in success, including motivation and reinforcement techniques.
**9. Health system or training gaps**	- Are there gaps in training or resources? - Tools or support that could improve practice? - Confidence in delivering interventions	Identify systemic issues and professional needs.
**10. Reflections on stigma**	- Does perceived stigma affect engagement? - Do clients hide smoking or under-report cigarettes? - How do advisors address stigma without alienating clients?	Highlight sensitive handling of judgment or shame; probe strategies to overcome stigma.
